# The Profiles of Tet-Mediated DNA Hydroxymethylation in Human Gliomas

**DOI:** 10.3389/fonc.2022.621460

**Published:** 2022-04-14

**Authors:** Aneta Brągiel-Pieczonka, Gabriela Lipka, Angelika Stapińska-Syniec, Michał Czyżewski, Katarzyna Żybura-Broda, Michał Sobstyl, Marcin Rylski, Marta Grabiec

**Affiliations:** ^1^ Department of Clinical Cytology, Centre of Postgraduate Medical Education, Warsaw, Poland; ^2^ Department of Neurosurgery, Institute of Psychiatry and Neurology, Warsaw, Poland; ^3^ Department of Radiology, Institute of Psychiatry and Neurology, Warsaw, Poland

**Keywords:** epigenetics, 5-hydroxymethylcytosine, ten-eleven translocation enzymes, brain tumors, glioblastoma

## Abstract

Gliomas are the most common primary malignant intracranial brain tumors. Their proliferative and invasive behavior is controlled by various epigenetic mechanisms. 5-hydroxymethylcytosine (5-hmC) is one of the epigenetic DNA modifications that employs ten-eleven translocation (TET) enzymes to its oxidation. Previous studies demonstrated altered expression of 5-hmC across gliomagenesis. However, its contribution to the initiation and progression of human gliomas still remains unknown. To characterize the expression profiles of 5-hmC and TET in human glioma samples we used the EpiJET 5-hmC and 5-mC Analysis Kit, quantitative real-time PCR, and Western blot analysis. A continuous decline of 5-hmC levels was observed in solid tissue across glioma grades. However, in glioblastoma (GBM), we documented uncommon heterogeneity in 5-hmC expression. Further analysis showed that the levels of TET proteins, but not their transcripts, may influence the 5-hmC abundance in GBM. Early tumor-related biomarkers may also be provided by the study of aberrant DNA hydroxymethylation in the blood of glioma patients. Therefore, we explored the patterns of TET transcripts in plasma samples and we found that their profiles were variously regulated, with significant value for *TET2*. The results of our study confirmed that DNA hydroxymethylation is an important mechanism involved in the pathogenesis of gliomas, with particular reference to glioblastoma. Heterogeneity of 5-hmC and TET proteins expression across GBM may provide novel insight into define subtype-specific patterns of hydroxymethylome, and thus help to interpret the heterogeneous outcomes of patients with the same disease.

## Introduction

The vast majority (80%) of malignant brain tumors is represented by gliomas (1). They have been classified by the World Health Organization into four grades, with Grade IV glioblastoma (GBM) as the most aggressive form. From 2016 the WHO grading is based on histological and molecular characteristics that are observed amongst various stages of gliomas (2). Many studies focused on genomic or transcriptomic profiles of human gliomas demonstrated that although histologically similar, they constitute distinct subtypes (3–5) that are associated with different survival outcomes (6, 7). Therefore, systematic molecular analysis of human gliomas is essential for their comprehensive understanding. Aberrations in genes and molecular pathways, include *IDH1/IDH2* and *H3.1/H3.3* mutation or loss of *TP53* tumor suppressor gene, can be used together with histopathological findings for gliomas classification (8, 9).

However, dysregulation of epigenomes seems to be the primary molecular mechanism involved in the pathogenesis of human gliomas. Epigenetic alterations, such as modifications of DNA (10, 11) and histones (12, 13), nucleosome remodeling (14, 15) and RNA-mediated silencing (16–18) are pointed out as a source of gliomas phenotypic heterogeneity. DNA methylation is the best-studied epigenetic change in this field (19–21), but some reports revealed the existence of other modifications in DNA methylome.

The ten-eleven translocation (TET) enzymes can alter DNA methylation status by converting 5-methylcytosine (5-mC) to 5-hydroxymethylcytosine (5-hmC), and later to 5-formylcytosine (5-fC) and 5-carboxylcytosine (5-caC) (22). 5-hmC may act as a transient intermediate in the process of 5-mC demethylation, as well as, may epigenetically regulate gene expression. Several reports found the relationship between 5-hmC level and glioma grades (10, 23–26). However, 5-hmC modification and its direct effect on gliomas biology need to be investigated.

Here, we picture the TET-dependent hydroxymethylation patterns in human gliomas. Using solid tumor tissues samples we demonstrate heterogeneity in 5-hmC expression across glioblastoma which can provide novel insight into define subtype-specific patterns of 5-hmC, and thus help to interpret the heterogeneous outcomes of patients with the same disease.

## Materials and Methods

### Clinical Samples

34 pairs of matched glioma tissues and blood samples were collected during standard neurosurgical tumor removals at the Department of Neurosurgery, Institute of Psychiatry and Neurology (Warsaw, Poland). Additionally, five independent random controls of blood samples were obtained from healthy volunteers. All solid tissues were submerged in the stayRNA solution (A&A Biotechnology) and stored at –80°C. Blood was collected into EDTA-treated tubes and centrifuged at 3500 rpm (MPW, Centrifuge MPW-350R, Rotor 1236B) for 10 min at 4°C. Then supernatant (plasma) was immediately transferred into the clean Eppendorf tubes and stored at –80°C.

### 5-hmC Quantification

The absolute level of 5-hmC in genomic DNA, previously extracted from 34 glioma tissues, was estimated with the EpiJET 5-hmC and 5-mC Analysis Kit (ThermoFisher Scientific). Briefly, 100 ng of DNA was glucosylated by T4 phage β-glucosyltransferase (T4 BGT), followed by subsequent digestion with Epi MspI and Epi HpaII enzymes. Then the percentage of cytosine modifications within CCGG sites was determined by quantitative real-time PCR (qRT-PCR) with a primer pair flanking recognition sequence. Primer sequences were as follows:

primer1_forward 5′-CTGTCATGGTGACAAAGGCATC-3′,

primer2_reverse 5′-CAGGATTTCTCTATTATGAAGACCTTG-3′.

The experiment was run in triplicate and the amount of 5-hmC was calculated as a percentage based on controls included in the kit.

### Quantitative Real-Time PCR

Following Chomczynski`s protocol (27), total RNA of glioma tissues was extracted by TRIzol Reagent (Life Technologies), whereas Total RNA Mini Concentrator Kit (A&A Biotechnology) was used for the extraction of RNA from plasma samples. The concentration and purity of RNA samples were assessed by measuring the 260/280 ratio of absorbance values with the Synergy H4 spectrophotometer (BioTek). cDNA was synthesized from 500 ng of RNA using random hexamers and TaqMan Reverse Transcription Reagents (ThermoFisher Scientific) according to the manufacturer`s instructions. Transcript levels of *TET* family genes were determined by the quantitative real-time PCR using 5x HOT FirePolEvaGreen qPCR Mix (Solis Biodyne) and primer sets for *TET1*, *TET2*, *TET3*, and the housekeeping gene *GAPDH*. The primer sequences are listed in **Supplementary Table S1**. All samples were run in triplicate, and data were normalized to the expression of *GAPDH* (28), according to the ΔCt method. While the ΔΔCt method was applied for relative quantification in blood samples.

### Western Blot

Thirty-four human glioma tissues were homogenized with TissueLyser LT homogenizer (Qiagen) in lysis buffer containing 2% SDS, pH 6.8 and protease inhibitors (Sigma). According to the protocol of Ericsson et al. (29), homogenates were incubated at 70°C and shaking at 1400 rpm (BioSan, Thermo-Shaker TS-100) for 10 min and then centrifuged at 12000 rpm (Eppendorf, Centrifuge 5415R, Rotor F45-24-11) for 5 min. The concentration of protein extracts was determined with the Bradford protein assay (Sigma). 15 µg of each protein sample was separated with 7% SDS-polyacrylamide gels and transferred onto nitrocellulose membranes using the Bio-Rad MINI Protean system. Immunoblotting with primary antibodies against TET1, TET2, TET3 (1:3000, ThermoFisher Scientific) and GAPDH (1:5000, Millipore) was performed overnight at 4°C, whereas secondary antibodies (a-mouse and a-rabbit, Vector) diluted 1:10000 were incubated with the membranes for one hour at room temperature. Blots were visualized with the WesternBright Quantum detection system (Advansta) on the UVITEC Cambridge scanner. Densitometry analysis was conducted using GelAnalyzer 2010 software.

### Statistical Analysis

GraphPad Prism 7.02 software was used to analyze the data. Statistical significance of differences between groups was determined by One-way ANOVA or nonparametric Kruskal-Wallis tests followed by *post hoc* Tukey or Dunn analysis, as well as with Mann-Whitney U test (*p < 0.05, **p < 0.01, ***p < 0.001). The results are presented as the mean ± standard deviation of the mean (SD).

## Results

### Global Changes in 5-hmC Abundance in Gliomas

So far, decreased 5-hmC levels have been presented in a variety of tumors (30–32), suggesting that the loss of 5-hmC may be considered as an epigenetic hallmark of the disease. To evaluate the global changes in 5-hmC abundance in human brain tumors, we selected DNA of 34 glioma samples represented by different WHO grades and analyzed them with EpiJET 5-hmC and 5-mC Analysis Kit and quantitative real-time PCR. Clinical and epigenetic characteristics of glioma samples are shown in **Table 1**.

**Table 1:** Characteristics of glioma patients.

Total cases = 34				
WHO grade	II	III	IV-low _5-hmC_	IV-high _5-hmC_
Number of patients	6	7	15	6
Age at diagnosis (years)				
Mean	39.3	41.4	65.9	63.2
Range	24 - 54	26 - 59	53 - 77	52 - 77
Gender				
Male/Female	3/3	4/3	9/6	3/3
Hemisphere				
Left/Right	1/5	4/3	8/7	1/5
Location				
Frontal	2	2	5	–
Temporal	3	5	4	4
Pariental	1	–	2	–
Occipital	–	–	4	2
Tumor status				
Primary/Recurrent	5/1	6/1	15/0	5/1
*IDH1* status				
*IDH1* wild-type	6	6	15	6
*IDH1* mutant	0	1 (R132G)	0	0
5-hmC (%)	8.2	3.5	3.0	20.0

**Figure 1:**
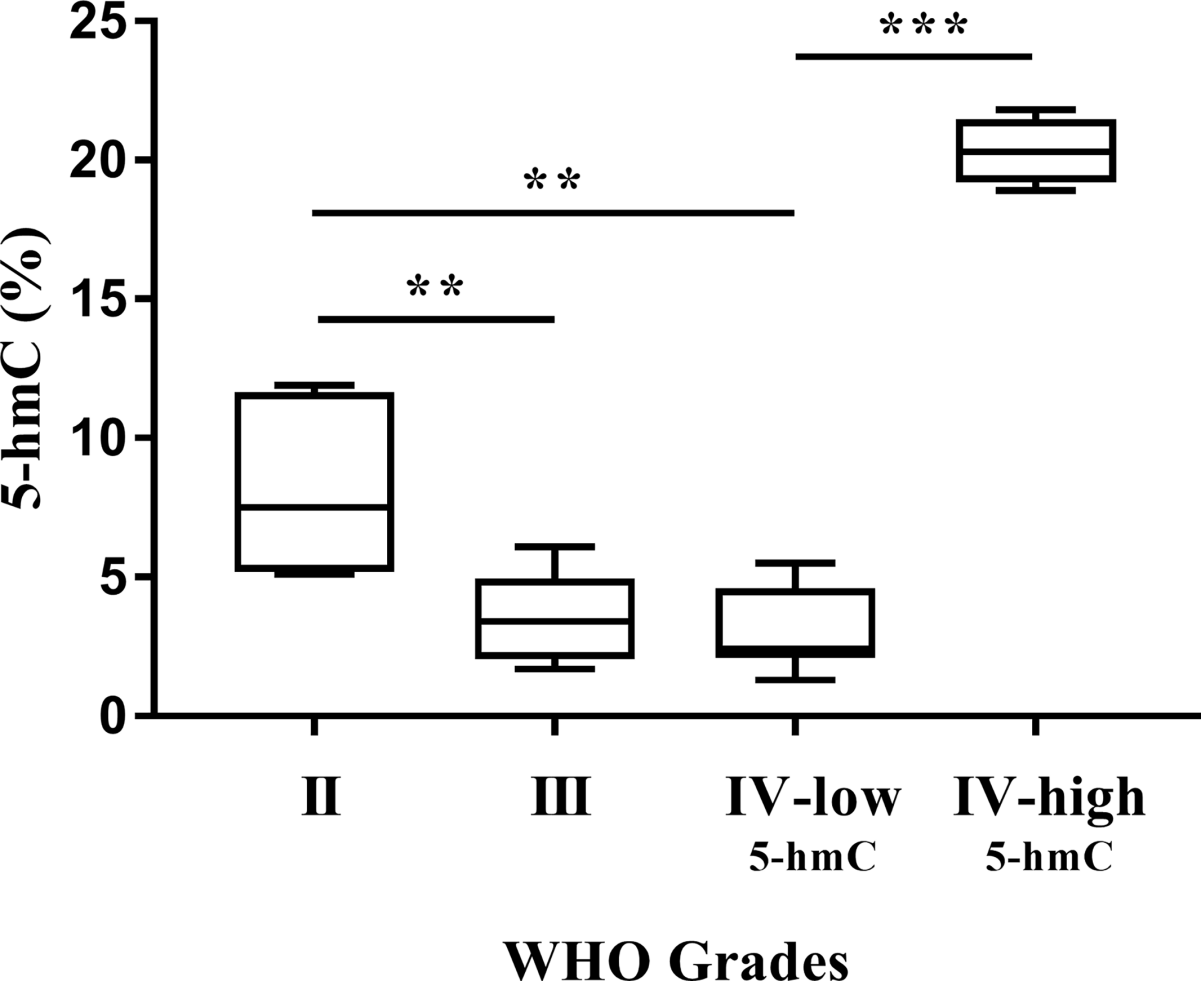
Global 5-hmC abundance (%) in gliomas. Increasing WHO grades of glioma are associated with a continuous decline in 5-hmC levels, except for glioblastoma (IV-low_5-hmC_ and IV-high_5-hmC_). Differences among group means were evaluated by one-way ANOVA test (**p < 0.01, ***p < 0.001).

As can be seen in **Figure 1** and **Table 1**, gliomagenesis has generated the changes of DNA hydroxymethylation. The 5-hmC expression correlated negatively with WHO grading, ranged from 8.2% in Grade II gliomas to 3.0% in Grade IV-low_5-hmC_ GBM. Significant differences were observed between Grades II and III (p < 0.01), as well as Grades II and IV-low_5-hmC_ (p < 0.01). While we observed a decreasing trend in 5-hmC expression with a higher tumor stage, we documented a strong elevation in 5-hmC level in around 30% of Grades IV GBM (Grade IV-high_5-hmC_). In this study group, the 5-hmC level was 20% and was significantly higher than in Grade IV-low_5-hmC_ samples (3%, p < 0.001). To our knowledge, this is one of the few reports describing high variability in 5-hmC abundance at the tumor mass (10, 24). To clarify observed diversity, the GBMs samples were categorized according to their *IDH1*, and developmental status. The results had not revealed molecular differences between both categories in this scope. A great majority of samples (95%) were primary glioblastomas, while all of them demonstrated the absence of mutation in *IDH1* gene.

Recognized differences might be a consequence of alterations in the expression of TET enzymes that catalyze the conversion of 5-mC to 5-hmC.

### TET Expression in Gliomas

**Figure 2:**
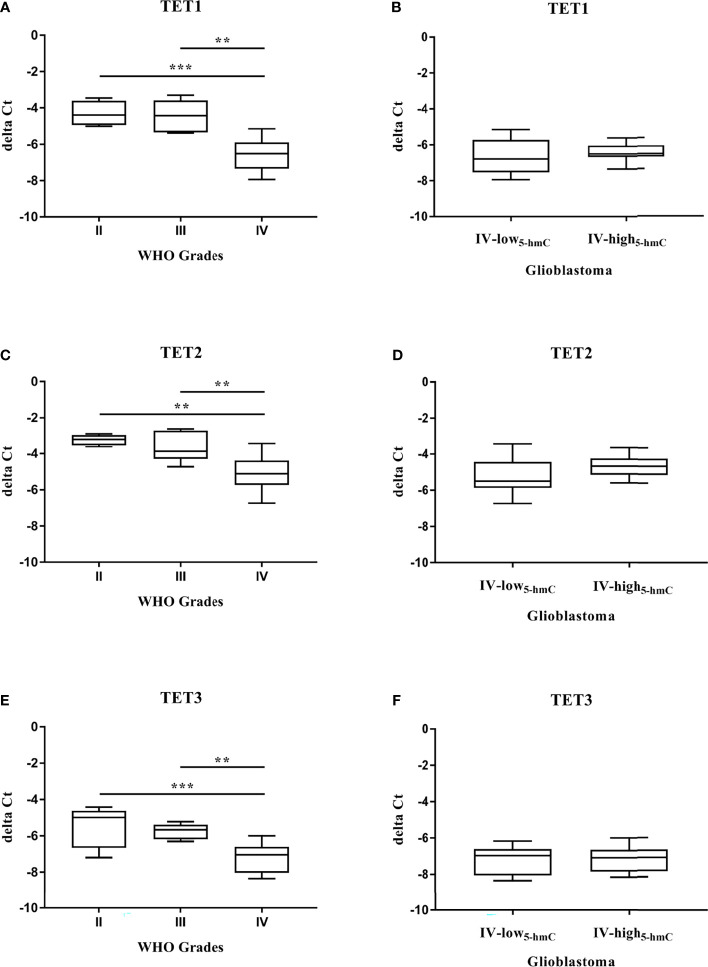
Quantitation of *TET* transcripts in gliomas. qRT–PCR analysis of *TET* mRNA **(A–F)**. The expression of *TET1* **(A)**, *TET2* **(C)**, *TET3* **(E)** mRNA significantly decreased at a higher WHO grade of glioma. Differences among group means were evaluated by one-way ANOVA test (**p < 0.01, ***p < 0.001). High variability in 5-hmC abundance across glioblastoma (Grade IV-low_5-hmC_ and Grade IV-high_5-hmC_) was not linked with levels of *TET1* **(B)**, *TET2* **(D)**, *TET3* **(F)** transcripts.

**Figure 3:**
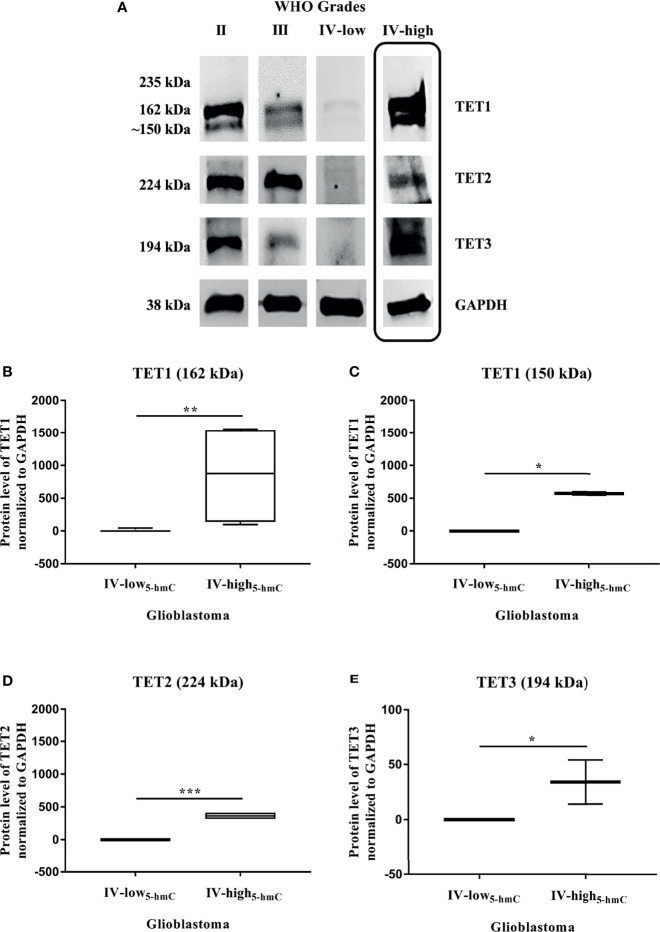
TET proteins expression in gliomas. Western blot analysis of TET proteins **(A–E)**. Exemplary immunoblots of TET1, TET2, and TET3 proteins pictured loss of their expression across glioma stages, except some Grade IV samples (last column). Expression levels of these proteins were normalized with GAPDH **(A)**. High variability in protein levels of TET1 **(B, C)**, TET2 **(D)** and TET3 **(E)** correlated with 5-hmC abundance across glioblastoma (Grade IV-low_5-hmC_ and Grade IV-high_5-hmC_). Differences between two groups were evaluated by Mann-Whitney U test (*p < 0.05, **p < 0.01, ***p < 0.001).

To define the impact of TET enzymes on changes in DNA hydroxymethylation, we analyzed their expression at the gene and protein levels in solid tumor tissues. Quantitative real-time PCR was performed to examine the mRNA expression of *TET* family genes (*TET1*, *TET2*, and *TET3*) in 34 samples, including 6 Grade II gliomas, 7 Grade III gliomas and 21 Grade IV GBM. We found that the relative mRNA levels of all three *TET* genes were strongly reduced during glioma grades (**Figures 2A, C, E**). Their downregulation was significantly higher in Grade IV GBM in comparison with Grade II gliomas (*TET1* and *TET3* p < 0.001, *TET2* p < 0.01) and Grade III gliomas (*TET1* p < 0.001, *TET2* and *TET3* p < 0.01). As the decline in *TET* mRNA was associated with glioma grades and 5-hmC expression, we determined the levels of *TET* transcripts in two groups with high variability in total 5-hmC abundance across GBM (Grade IV-low_5-hmC_ and Grade IV-high_5-hmC_, **Figure 1**). There were no significant differences in *TET1 (p = 0.1322)*, *TET2 (p = 0.2434)* and *TET3 (p = 0.8208)* mRNA levels between glioblastoma Grade IV-low_5-hmC_ and Grade IV-high_5-hmC_ (**Figures 2B, D, F**). Next, we evaluated the levels of TET proteins in the same samples by Western blot. Expression levels of each TET proteins are shown in **Supplementary Table S2**. Immunoblotting revealed the absence of TET1 isoform at 235 kDa, which is mainly detected in embryonic stem cells. But, two additional bands at 162 kDa and 150 kDa were found in the majority of instances. TET2 and TET3 isoforms were discovered at 224 and 194 kDa, respectively (**Figure 3A**). As in the case of *TET* transcripts expression (**Figures 2A, C, E**), levels of TET proteins were negatively associated with advanced stages of gliomas. From Grade II gliomas to Grade IV GBM, we noticed 2-, 4- and 6-fold reduction in TET1_162 and 150 kDa_, TET2_224 kDa_ and TET3_194 kDa_ levels, respectively. Interestingly, in Grade IV samples, we observed high variability in TET isoforms expression pattern, which could affect 5-hmC abundance. Quantification of normalized values showed significant differences in TET1_162 and 150 kDa_ (p < 0.01, p < 0.05), TET2_224 kDa_ (p < 0.001) and TET3_194 kDa_ (p < 0.05) protein levels between GBM Grade IV-low_5-hmC_ and Grade IV-high_5-hmC_ (**Figures 3B**–**E**). These findings suggested the potential role of TET proteins patterns in setting the 5-hmC level in Grade IV glioblastoma.

### TET Transcripts Profiling in Plasma Samples

**Figure 4:**
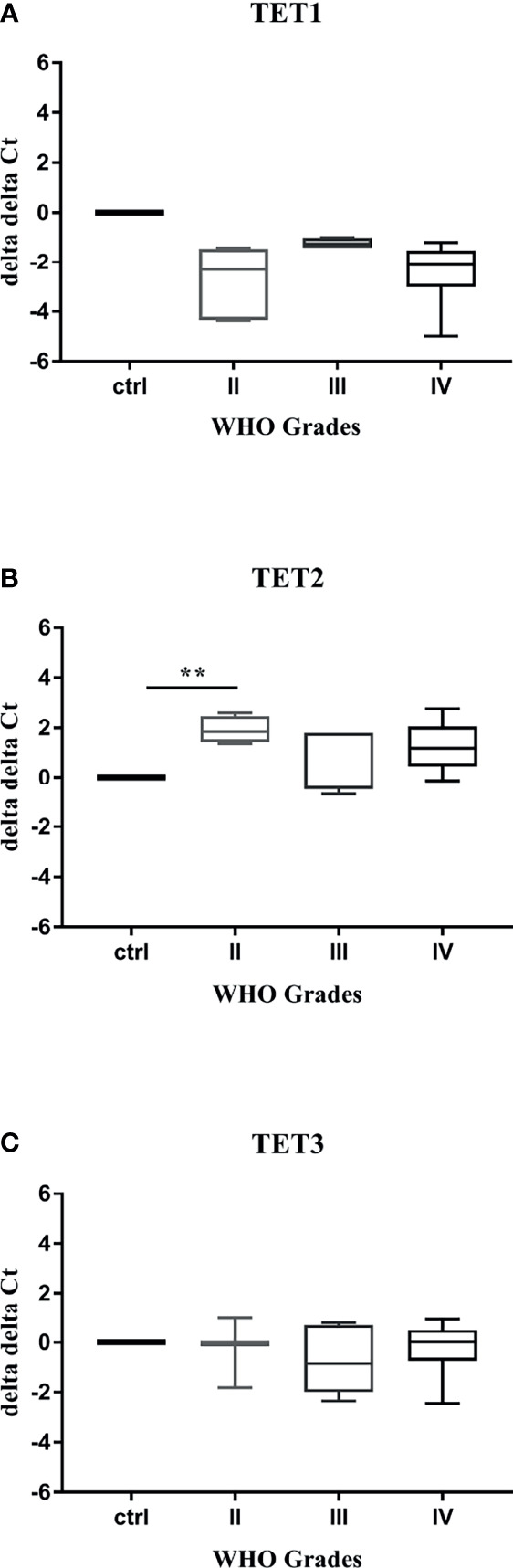
*TET* transcripts profiling in plasma samples. qRT–PCR analysis of *TET* mRNA **(A–C)**. Levels of *TET1* **(A)**, *TET2* **(B)**, and TET3 **(C)** transcripts were variously regulated in plasma samples obtained from patients with gliomas. Statistical analysis performed using non-parametric Kruskal-Wallis test (**p < 0.01).

The prospect of distinguishing the aberrant DNA hydroxymethylation in the blood of glioma patients may indicate a powerful tool for early cancer detection or monitoring its progress. To explore the potential diagnostic features of *TET* transcripts profiling, we performed the quantitative real-time PCR on 34 plasma samples obtained from patients with Grade II or III or IV gliomas, and 5 healthy controls. Expression of *TET* genes was observed in the majority of examined samples, followed by specific numbers for *TET1* (control: 80% vs. Grade: II-83%, III-57%, IV-70%), *TET2* (control: 80% vs. Grade: II-83%, III-71%, IV-91%) and *TET3* (control: 80% vs. Grade: II-50%, III-86%, IV-91%) transcript. Our results confirmed that the employed method was sensitive to low-input mRNA presented in plasma samples. The further analysis evaluated the relative mRNA levels of *TET* genes in plasma samples obtaining from patients with different WHO grades gliomas and compared them to healthy controls (**Figures 4A**–**C**). The results displayed various profiles for each transcript. While *TET1* characterized slightly lower expression than control, *TET3* was similar to control values. The expression of both transcripts was unaffected by WHO grading (**Figures 4A, C**). Whereas, the level of *TET2* was significantly increased in plasma samples derived from patients with Grade II gliomas compared to controls (p < 0.01, **Figure 4B**). Validation of our preliminary results in a larger population of patients is needed to evaluate their use as potential biomarkers for early-stage gliomas diagnostics.

## Discussion

Currently, the molecular landscape of brain tumors is described by epigenetic mechanisms include DNA methylation and hydroxymethylation (10, 11), histone modifications (12, 13), nucleosome remodeling (14, 15) and RNA-mediated silencing (16–18), that may clarify their etiologic evolution. Recently rediscovered, oxidized form of 5-methylcytosine (5-hmC) may act as a transient intermediate in the process of 5-mC demethylation or may epigenetically mark the cellular state itself with different biological roles.

In the present study, we characterize the epigenetic profile of DNA hydroxymethylation in human gliomas. Decreased level of 5-hmC was observed in glioma patients compared to healthy controls (33–35), but also it was related to glioma grades (10, 23–26). Our results confirm that the loss of 5-hmC is a hallmark of high-grade gliomas. However, we documented a significant increase of global 5-hmC abundance in almost a third of Grades IV GBM. This is one of the few findings that demonstrated heterogeneity in 5-hmC expression across the bulk of the glioblastoma (10, 24). According to recent research, the intra-tumoral diversity of 5-hmC levels among single GBM cells, that represent the proliferative, stem-like and tumorigenic states, could clarify unexpected results (36). But, dysregulation of TET enzymes function may be a possible biological explanation for the observed variability as well. The influence of TETs, including their genetic alterations and subcellular localization, on 5-hmC status in glioma cells, was described by several studies (23–25, 37, 38). Recently, epigenetic repression of histone marks (H4K16ac, H3K4me3, H3K9ac, H3K36me3, H3K4me1, H3K27ac) in *TET3* gene has been postulated as a driver of glioblastoma development *via* genome-wide alteration of 5-hmC (39). Generally, it is believed that the decline in *TET* genes expression causes a widespread reduction of 5-hmC and poorer prognosis in glioma patients. We found that *TET1*, *TET2*, *TET3* mRNA, and 5-hmC levels were decreased during glioma grades. However, similarly to Jin and Glowacka results (40, 41), there was no correlation between expression of *TET* transcripts and variability in 5-hmC abundance across the bulk of the glioblastoma. To define the complete impact of TETs on changes in DNA hydroxymethylation, we evaluated their protein levels in the same samples. Proteins produced by the TET2 and TET3 isoforms were expressed as expected bands at 224 kDa and 194 kDa. Instead of the 235 kDa canonical TET1 protein, we found two short isoforms (162 kDa and ~150 kDa). According to previous reports, they are exclusively activated from an alternate promoter in somatic cancer cells (42, 43). The levels of TET proteins, just as *TET* transcripts, were negatively associated with high-grade gliomas. However, Grade IV GBM samples were more variable in this field. Significant heterogeneity in the expression of TET2 protein in glioblastoma was also noted by Briand (44). We furthermore pointed out the relation between variable levels of TET proteins and 5-hmC abundance across glioblastoma. Our findings suggest that the regulation of TET transcription and translation can be made in a different way. For example, this observed imbalance may be a result of different actions of transcription factors, RNA binding proteins, miRNAs targeting mRNA or post-translational modifications (like phosphorylation, acetylation, glycosylation, etc.). Based on the patient-derived glioma stem cells (GSCs) model, the transcription factor (SOX2)-oncomiR (miR-10b-5p)-TET2 axis was identified, which plays an important role in promoting GBM oncogenesis (45). However, it is one of many potential mechanisms involved in glioblastoma growth.

In summary, we demonstrated that expression patterns of TET proteins and the 5-hmC abundance are changed in Grade IV GBM, but the molecular mechanism of this process still needs to be clarified.

Recently, many studies on gliomas indicated the presence of circulating cell-free coding and non-coding nucleic acids in blood or other biofluid samples (46–48). Therefore, liquid biopsy has become a new diagnostic tool that may establish and track the stage of cancer with particular biomarkers. Cai’s group, for example, has developed a noninvasive 5-hmC detection method in circulating cell-free DNA of glioma patients, which was able to distinguish the difference between GBM and lower-grade gliomas regardless of *IDH1* mutation status (49). Here, we explore the potential diagnostic features of *TET* transcripts in plasma samples obtained from patients with gliomas. All three *TET* transcripts were detected in plasma, but their profiles differed from those in solid tissue. We showed that the plasma relative mRNA level for *TET1* was decreased in every stage of glioma, while the *TET3* level remained unchanged. The most promising results were provided by *TET2* gene that was significantly increased in Grade II glioma. Validation of our preliminary results in a larger population of patients is needed to evaluate their use as potential biomarkers for glioma diagnostics.

To conclude, we found that global abundance of 5-hmC was negatively correlated with glioma WHO grades and variable across the bulk of the glioblastoma. It was followed by various TET proteins patterns in solid tumor tissues. In contrast, profiles of *TET* transcripts in plasma samples displayed its heterogeneity. However, significant overexpression of *TET2* in Grade II gliomas might offer a new tool for effective diagnosis of lower-grade glioma patients. Our findings provide novel information about the potential role of TET epigenetic regulation in human gliomas, with particular reference to glioblastoma.

## Data Availability Statement

All data generated during this study are included in the article. Further inquiries can be directed to the corresponding author.

## Ethics Statement

The study was conformed to the standards set by the Declaration of Helsinki and approved by the ethics committee of the Centre of Postgraduate Medical Education (code: 34/PB/2017). Written informed consent was obtained from participants prior to specimen collection.

## Author Contributions

MG designed all experiments, interpreted the results, and wrote the manuscript. AB-P, GL performed the experiments, analyzed the data. KŻ-B performed part of molecular analysis. AS-S, MC contributed to sample collection and registered clinical information. MS and MR managed sample collection; critically commented on the manuscript. All authors read and approved the final version.

## Funding

This study was supported by a statutory grant 501-1-027-03-19 from the Centre of Postgraduate Medical Education in Warsaw.

## Conflict of Interest

The authors declare that the research was conducted in the absence of any commercial or financial relationships that could be construed as a potential conflict of interest.

## Publisher’s Note

All claims expressed in this article are solely those of the authors and do not necessarily represent those of their affiliated organizations, or those of the publisher, the editors and the reviewers. Any product that may be evaluated in this article, or claim that may be made by its manufacturer, is not guaranteed or endorsed by the publisher.
