# Supplementary figures and images for: The Profiles of Tet-Mediated DNA Hydroxymethylation in Human Gliomas
Source: Front Oncol. 2022 Apr 14;12:621460. doi: 10.3389/fonc.2022.621460 (PMC9047681; doi:10.3389/fonc.2022.621460)

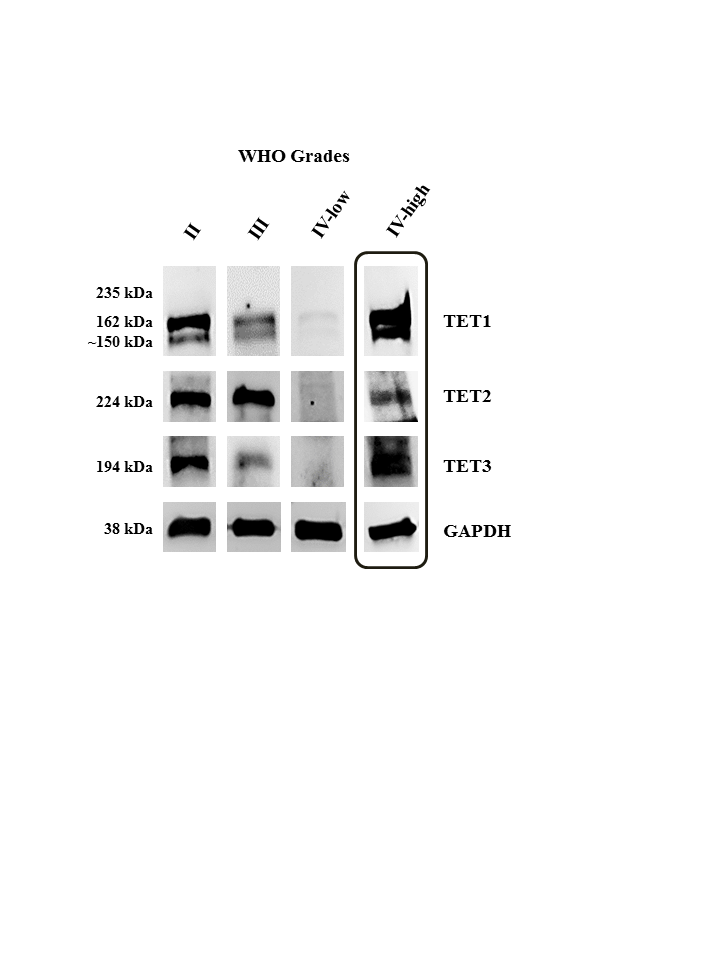

Supplement: Supplementary file 1 [file Image_1.tiff]
