# Supplementary material for: The Profiles of Tet-Mediated DNA Hydroxymethylation in Human Gliomas
Source: Front Oncol. 2022 Apr 14;12:621460. doi: 10.3389/fonc.2022.621460 (PMC9047681; doi:10.3389/fonc.2022.621460)
Supplement: Supplementary file 2 [file Table_1.doc]

**Additional file 1**

**TABLE S1** Primers used in qRT-PCR.

| **Gene** | **Product length (bp)** | **Primer**  **forward or reverse** | **Sequence (5`- 3`)** |
| --- | --- | --- | --- |
| TET1 | 141 | F | CAGAACCTAAACCACCCGTG |
|  |  | R | TGCTTCGTAGCGCCATTGTAA |
| TET2 | 95 | F | GATAGAACCAACCATGTTGAGGG |
|  |  | R | TGGAGCTTTGTAGCCAGAGGT |
| TET3 | 169 | F | TCCAGCAACTCCTAGAACTGAG |
|  |  | R | AGGCCGCTTGAATACTGACTG |
| GAPDH | 101 | F | CTGGGCTACACTGAGCACC |
|  |  | R | AAGTGGTCGTTGAGGGCAATG |
