# Supplementary material for: The Profiles of Tet-Mediated DNA Hydroxymethylation in Human Gliomas
Source: Front Oncol. 2022 Apr 14;12:621460. doi: 10.3389/fonc.2022.621460 (PMC9047681; doi:10.3389/fonc.2022.621460)
Supplement: Supplementary file 3 [file Table_2.doc]

**Additional file 2**

**TABLE S2** Expression levels of TET proteins.

|  | **WHO Grades** | | | |
| --- | --- | --- | --- | --- |
|  | **II** | **III** | **IV-low** | **IV-high** |
| **TET1** |  |  |  |  |
| 235 kDa | not observed | | | |
| 162 kDa | 100%  **/*** | 100%  **/*** | 38%  * | 56%  **/*** |
| ~150 kDa | 100%  */** | 71%  ** | 25%  * | 45%  **/*** |
| **TET2** |  |  |  |  |
| 224 kDa | 83%  **/*** | 86%  ** | 12%  * | 67%  **/*** |
| **TET3** |  |  |  |  |
| 194 kDa | 67%  */** | 43%  ** | 0% | 34%  * |

*-*** scale of TETs expression intensity

(%) number of the positive signals at GBMs samples that represented different WHO Grades
